# Structural and microstructural changes in white and gray matter across the Alzheimer’s disease continuum

**DOI:** 10.3389/fnagi.2025.1693840

**Published:** 2025-11-14

**Authors:** Lingling Lv, Hui Guo, Zhiru Zhao, Xiongfei Zhao

**Affiliations:** Department of Neurology, Xianyang Hospital of Yan’an University, Xianyang, Shanxi, China

**Keywords:** Alzheimer’s disease, gray matter, white matter, diagnosis, mild cognitive impairment

## Abstract

Alzheimer’s disease (AD) is a neurodegenerative disorder characterized by progressive brain atrophy, with pathological progression accompanied by significant structural alterations in both gray matter (GM) and white matter (WM). This review summarizes the neuroimaging features and clinical implications of brain volumetric changes across distinct the clinical phases of the AD continuum [preclinical phase, subjective cognitive decline (SCD), mild cognitive impairment (MCI), and dementia phase]. Our analysis reveals a key conceptual advance: the spatiotemporal pattern of WM volume loss is not merely a consequence of GM degeneration but an active and complementary contributor to clinical decline. We identify specific, underappreciated WM tracts whose atrophy rates offer unique prognostic value beyond hippocampal volume. The primary contribution of this work is a unified model of AD neuroanatomy, which challenges the isolated view of GM and WM pathology. This refined understanding is critical for developing the next generation of biomarkers and underscores the imperative to leverage artificial intelligence for analyzing these complex, multi-tissue interactions. Future research should further integrate artificial intelligence and multi-omics data to refine personalized predictive models.

## Introduction

**FIGURE 1:**
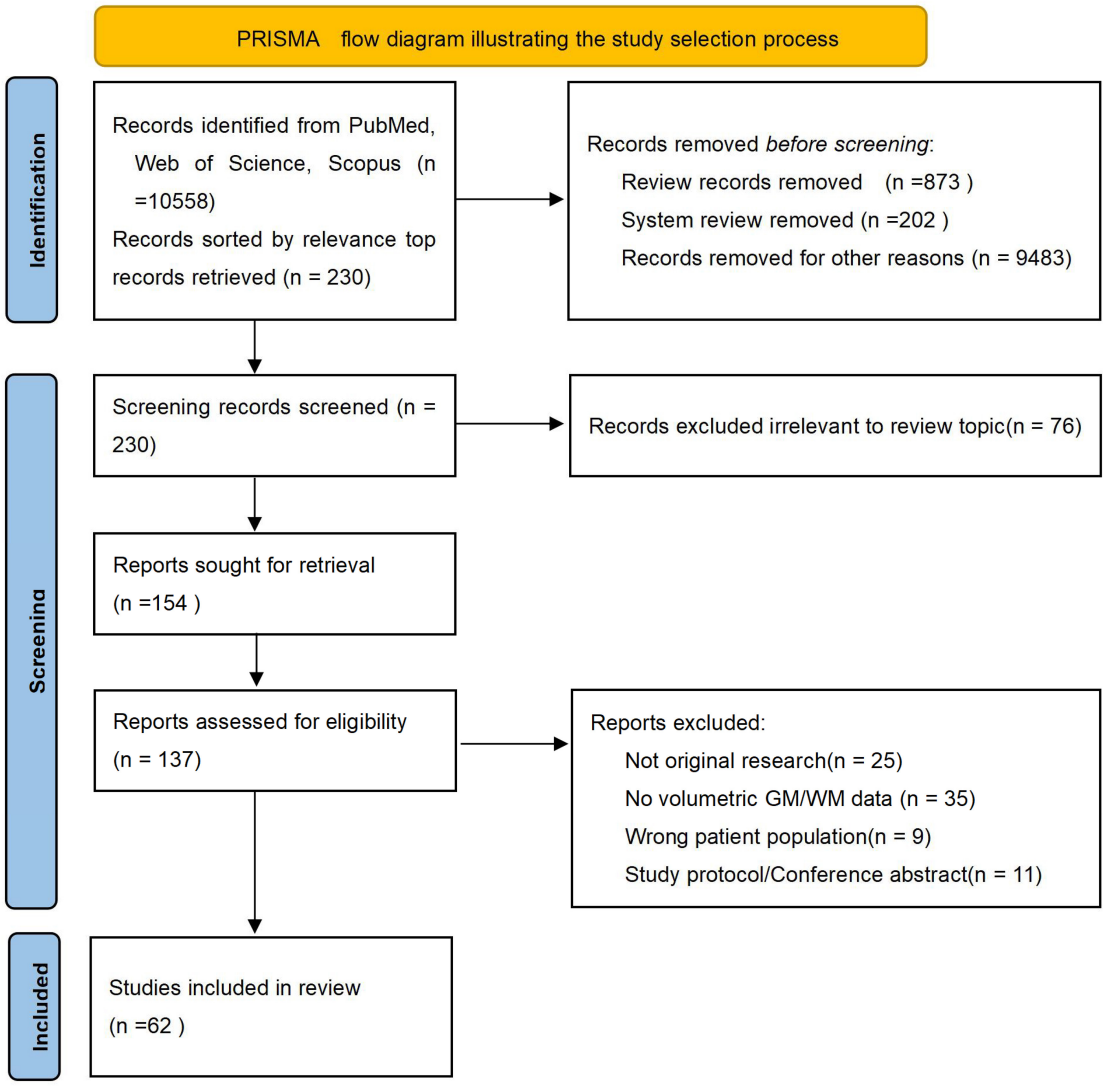
PRISMA flow diagram illustrating the study selection process.

Alzheimer’s disease (AD), recognized as the predominant cause of dementia, has emerged as one of the most lethal and burdensome diseases of the 21st century (Alzheimers Dement, 2024; Scheltens et al., 2021). The disease progresses along a continuum, encompassing the preclinical phase, subjective cognitive decline (SCD), mild cognitive impairment (MCI), and the dementia phase (Jack et al., 2024). The hallmark neuropathological features of AD–amyloid-β plaques and neurofibrillary tangles–drive a progressive neurodegenerative process, which is captured *in vivo* by magnetic resonance imaging as gray matter (GM) atrophy (Jack et al., 2018). Notably, brain atrophy in AD is detectable at early clincial phase, particularly in limbic structures and the gyri of the frontal and temporal cortices, offering valuable insights into disease progression (Dickerson et al., 2011). This review synthesizes evidence from medium- to high-quality clinical studies published between 2019 and 2024 that concurrently examine structural changes in both GM and white matter (WM) across the AD continuum. To ensure comprehensive coverage, we systematically searched PubMed, Web of Science Core Collection, Scopus, and Embase using the following core search terms and their variants: “gray matter,” “white matter,” “Alzheimer’s disease,” “Alzheimer disease,” combined with “MRI,” “volumetric,” “atrophy,” and “neuroimaging.” The search strategy was designed to capture all relevant studies investigating GM and WM alterations in AD spectrum disorders. In addition, the reference lists of relevant review articles were manually screened for potentially eligible publications. Due to the large volume of search results, a pragmatic screening strategy was adopted to focus on the most influential literature. Search outputs were sorted by relevance, and the top 230 records underwent further screening. The study selection process adhered to the PRISMA guidelines, as summarized in Figure 1. Following title and abstract screening, 76 records were excluded. Of the 154 full-text articles sought, 17 were unavailable, leaving 137 for eligibility assessment. After full-text review, 75 articles were excluded with documented reasons (e.g., not original research, lack of volumetric data), resulting in 62 studies included in the qualitative synthesis. This review aims to synthesize evidence from medium- to high-quality clinical studies published in the past 5 years that concurrently investigate structural alterations in both GM and WM across the AD continuum. Furthermore, this article will also specifically highlight recent advances in brain imaging technologies and their role in deciphering the mechanisms underlying AD-related structural changes.

## Studies on GM

In individuals with AD, the morphology, volume, and microstructure of GM exhibit profound degenerative changes. These alterations collectively underlie the core clinical manifestations of the disease, including memory impairment, cognitive dysfunction, and behavioral disturbances. GM degeneration serves as both a defining neuropathological feature of AD and a pivotal biomarker for tracking disease progression.

### Studies on the preclinical phase

Accelerated normal aging may facilitate the early detection of AD signs in healthy individuals (Avelar-Pereira et al., 2024). The preclinical detection of AD can be achieved through the integration of neuroimaging markers and plasma biomarkers, with temporal lobe atrophy progression serving as a particularly sensitive indicator of impending cognitive decline (Mitolo et al., 2024). Standardized volumes of the entorhinal cortex showed a change of more than 20% up to 15 years prior to the onset of cognitive decline (Platero, 2022). Thus, establishing early detection systems will be a pivotal breakthrough in advancing AD research.

### Studies on SCD

Subjective cognitive decline serves as a critical indicator in the early detection and diagnosis of AD (Jessen et al., 2023). Although clinical deficits are absent, SCD may represent a preclinical phase characterized by neuroanatomical changes that are similar, albeit more subtle, to those observed in patients with amnestic MCI (aMCI) or AD dementia (Rivas-Fernández et al., 2023). Cortical and subcortical morphological changes may help preserve cognitive function through compensatory mechanisms in SCD (Yang et al., 2023). The dorsal precuneus, known to be associated with early AD, exhibits pronounced neuroimaging changes in individuals with SCD (Li X. Y. et al., 2024). Compared to the healthy control (HC) group, patients with SCD displayed relatively minor surface morphological changes, predominantly localized to the insula and pars triangularis (Yang et al., 2023). Compared with HC, SCD showed morphological changes in the right inferior temporal gyrus (ITG) (Wu et al., 2023), right insula, and right amygdala (Song et al., 2024). Compared to the HC, the SCD group exhibited decreased cortical thickness in the right ITG (Wu et al., 2023). However, individuals with SCD demonstrated greater hippocampal atrophy, reduced cognitive and functional performance, and more pronounced behavioral symptoms compared to the HC (Jessen et al., 2023). The Crus I region in the right cerebellum may serve as a potentially useful brain region for distinguishing progession SCD (SCDp) from non-progession SCD (SCDnp) (Deng et al., 2024). There were no significant differences in GM volume between individuals with SCD and HC (Serra et al., 2023).

Voxel-based morphometry (VBM) revealed GM atrophy in the middle frontal gyrus, superior orbital gyrus, superior frontal gyrus, right rectus gyrus, entire occipital lobe, thalamus, and precuneus in the SCD group (Riverol et al., 2024). Compared with HC, both SCD and MCI showed reduced left parietal lobe (IPL) volume. Both SCD and MCI groups showed reduced ReHo values and reduced GM volume in the right middle temporal gyrus compared with HC (Wu et al., 2023). Region of Interest (ROI) analysis showed volume reduction in the left rectus gyrus, bilateral medial orbital gyrus, middle frontal gyrus, superior frontal gyrus, calcarine fissure, and left thalamus (Riverol et al., 2024). SCDp showed greater hippocampal atrophy than SCDnp and controls. However, in the VBM analysis, the SCDp group only showed more hippocampal atrophy than the SCDnp group (Riverol et al., 2024). Compared with HC, SCD showed microstructural changes in the right ITG, lateral occipital, and insular fiber tracts (Song et al., 2024). Compared to the HC, the SCD group exhibited higher reduced fractional Amplitude of Low-Frequency Fluctuation (fALFF) and ReHo values in the left inferior occipital gyrus, reduced fALFF values and elevated functional connectivity values in the IPL (Wu et al., 2023). Patients with amyloid-β positive SCD (Aβ+SCD) experience greater cognitive decline and more pronounced medial temporal lobe atrophy within a 24-months period (Hong et al., 2023). The brain atrophy in SCD group was mainly in frontal lobe and occipital lobe. However, only the SCDp group showed medial temporal lobe atrophy at baseline (Riverol et al., 2024). The multimodal MRI combined with machine learning classification method has good performance in the classification of SCD caused by AD, which has the potential for clinical application. The diagnostic accuracy of SCD plus individuals was 79.49% in the Chinese cohort and 83.13% in the ANDI cohort (Lin et al., 2023). The prediction of early AD can be comprehensively assessed through a combination of MRI technology and clinical indicators (Devanarayan et al., 2024).

### Studies on MCI

Compared to the HC, patients with MCI primarily exhibited surface morphological changes in the left brain, including the transverse temporal gyrus, superior temporal gyrus, insula, and operculum (Yang et al., 2023). These observed morphological changes were significantly associated with clinical ratings of cognitive decline (Yang et al., 2023). Compared with MCI that transformed into AD, the width of multiple brain grooves in bilateral temporo-occipital region and left frontal region have significant changes (Sighinolfi et al., 2024). A sample Mendelian randomization analysis confirmed a potential causal relationship between a higher neurotic polygenic risk score and a reduced inferior parietal surface area, as well as an increased risk of transformation in patients with aMCI (Li Q. et al., 2024).

Hippocampal volume has proven to be an effective biomarker for distinguishing between the HC, MCI, and dementia groups. Clinical studies have found that individuals with MCI exhibit a 14% reduction in hippocampal volume, while those with dementia show a 22% reduction compared to healthy individuals (Convit et al., 1997). Participants with higher levels of education (>13 years) demonstrated superior cognitive performance and larger hippocampal volumes. Midbrain and locus ceruleus volumes are associated with deficits in attention and executive function in MCI (Dutt et al., 2021). Participants in the MCI group showed smaller olfactory roi GMV, including significant reductions in piriform cortex, amygdala, entorhinal cortex, and left hippocampus, compared with SCD and HC. There is specific atrophy in the limbic/medial-temporal olfactory processing areas in MCI, and this degree of atrophy may predict early cognitive decline in AD (Jobin et al., 2023). However, another meta-analysis found structural changes early in the disease are most pronounced in the medial temporal lobe, particularly in the entorhinal cortex, which, along with the hippocampus, offers similar discrimination as the disease progresses. Notably, when it comes to predicting the conversion from MCI to AD, the entorhinal cortex demonstrates better predictive accuracy than other structures, including the hippocampus (Leandrou et al., 2018). Cognitive reserve modulates cortical structures only in the early phases of dementia (Serra et al., 2022). MCI can revert to normal cognition (NC) under certain conditions, indicating that some patients may experience a more favorable cognitive trajectory (Yu et al., 2024). The scientific community recognizes MCI as a pivotal transitional phase in AD pathogenesis, characterized by substantial clinical and neurobiological heterogeneity. Current evidence underscores the imperative for comprehensive multimodal evaluation-integrating advanced neuroimaging parameters [e.g., hippocampal volumetry, tau-Positron Emission Tomography (PET) imaging], validated fluid biomarkers (including CSF p-tau181 and plasma GFAP), and polygenic risk profiling-to enable: (1) accurate phenotypical classification, (2) reliable prognostication of conversion risk, and (3) stratification for targeted therapeutic interventions. This integrative approach establishes a robust evidence base for precision medicine paradigms in prodromal AD management.

### Studies on the dementia phase

Patients with AD exhibit cognitive changes within a few years after MRI shows signs of atrophy, providing important insights for the early identification of AD (Mofrad et al., 2021). Hippocampal volume features are effective in differentiating between early and late AD lesions (Ranjbar et al., 2019). AD patients had reduced bilateral hippocampal volume and hypoperfusion of bilateral temporoparietal and posterior midline structures compared with HC (Tai et al., 2020). Radiomic analysis of hippocampal texture shows promise in distinguishing the clinical progression of AD (Ranjbar et al., 2019). The ratio of hippocampus to cortex emerges as the most effective structural MRI (sMRI) biomarker for differentiating between subtypes of AD, aligning with the spatial distribution of tau pathology and predicting the rate of cognitive decline (Krajcovicova et al., 2019). The hippocampal volume was positively correlated with plasma Aβ42 and Aβ42/Aβ40, and negatively correlated with Aβ40, and P-tau181 and p-tau217 concentrations were negatively correlated with temporal GM volume and cortical thickness in AD (Mitolo et al., 2024). Precuneus atrophy in healthy individuals is associated with an increased amyloid load, indicating potential alterations in AD (Avelar-Pereira et al., 2024). In the context of AD as predicted by cerebrospinal fluid (CSF) and MRI findings, elevated baseline levels of pTau-181 were found to correlate with significant reductions in total GM volumes, particularly within targeted regions of the medial temporal lobe. These observations indicate that pTau-181 has the potential to serve as a valuable biomarker for forecasting brain atrophy and cognitive decline among cognitively unimpaired older adults in the future. This highlights its significance in early intervention strategies aimed at mitigating the progression of neurodegeneration (Dark et al., 2024). Emerging neuroimaging studies have demonstrated that the volumetric ratio between GM structures and their adjacent ventricular compartments serves as a reliable neuroimaging biomarker for detecting early-phase neurodegenerative changes (Hu et al., 2023).

Alzheimer’s disease is characterized by progressive GM atrophy, particularly in cholinergic regions such as the Nucleus basalis of Meynert (NbM), which shows significant volume loss in AD patients compared to HC, though this effect is less pronounced in MCI (Mieling et al., 2023). Widespread structural alterations extend to the hippocampus (reduced volume and hypoperfusion in temporoparietal regions) (Tai et al., 2020), caudate nucleus (Tentolouris-Piperas et al., 2017), and brainstem (Jacobs et al., 2022), with some changes emerging even in preclinical phases. Notably, the AD genetic risk score (AD-GRS) exhibits age-dependent associations with volume loss across 38 brain regions in middle-aged and older adults, highlighting the interplay between genetic susceptibility and neurodegeneration (Buto et al., 2024). Conversely, lifelong physical activity correlates with preserved volume in prefrontal and hippocampal regions, suggesting modifiable protective factors (Erickson et al., 2012). Further, postmenopausal women with cognitive complaints demonstrate accelerated GM loss (Conley et al., 2020), while frontal lobe structures (e.g., prefrontal cortex, anterior cingulate) are the strongest predictors of neuropsychiatric symptom progression in dementia (Boublay et al., 2020). The authors posit that the future integration of multimodal biomarkers with precision preventive medicine could revolutionize AD management by enabling early prediction and targeted intervention a decade or more before clinical symptoms emerge. This paradigm shift would transform AD therapeutics from reactive treatment to proactive prevention, potentially halting pathology at its preclinical phase.

To guide future efforts, we have identified the most sensitive biomarkers for each AD phase and benchmarked their performance with key quantitative indicators, including effect sizes and diagnostic accuracy, to provide a clear reference for the field (Table 1).

**TABLE 1:** Phase-specific structural MRI biomarkers in the AD continuum.

Disease phase	Most sensitive biomarkers	Effect size (Cohen’s d)	AUC	Primary utility
Preclinical AD	GM: entorhinal cortex thickness	−0.6 to −0.8	0.75–0.85	Predictive
	GM: hippocampal volume (CA1 subfield)	−0.5 to −0.7	0.70–0.80	Predictive
	WM: fornix mean diffusivity	0.7 to 0.9	0.80–0.85	Predictive
SCD	GM: hippocampal volume	−0.8 to −1.0	0.80–0.90	Predictive/confirmatory
	GM: middle temporal gyrus thickness	−0.6 to −0.8	0.75–0.85	Confirmatory
	WM: parahippocampal cingulum MD	0.8 to 1.0	0.82–0.88	Predictive/confirmatory
MCI	GM: hippocampal volume	−1.2 to −1.5	0.85–0.92	Confirmatory
	GM: posterior cingulate cortex thickness	−1.0 to −1.2	0.80–0.87	Confirmatory
	WM: superior longitudinal fasciculus MD	1.0 to 1.3	0.83–0.89	Confirmatory
AD dementia	GM: widespread cortical thinning	>−1.5	>0.95	Confirmatory
	GM: ventricular enlargement	> +1.8	>0.90	Confirmatory
	WM: WMH burden	> +1.2	>0.75	Confirmatory

AD, Alzheimer’s disease; GM, gray matter; WM, white matter; SCD, subjective cognitive decline; MCI, mild cognitive impairment; MD, mean diffusivity; WMH, WM hyperintensities; AUC, area under the receiver operating characteristic curve. Effect sizes (Cohen’s d) are approximate ranges derived from meta-analyses and key studies cited in this review, comparing each phase to cognitively normal controls. AUC values represent the accuracy for distinguishing the specified phase from cognitively normal controls or for predicting conversion to the next phase (Predictive utility). Predictive, primarily useful for forecasting progression to a more advanced phase; Confirmatory, primarily useful for supporting the diagnosis at the current phase.

## Studies on WM

White matter abnormalities manifest during the early phases of AD pathogenesis and may actively contribute to disease progression (Bozzali et al., 2016). Histology studies show that the brain’s WM architecture is highly complex, with up to 98% of the WM consisting of multiple fibers with crossing fiber orientations (Dewenter et al., 2023). WMH burden is associated with cognitive changes and early cognitive decline in healthy older adults (Kamal et al., 2023). WMH specifically contributes to cognitive decline in AD patients independent of amyloid deposition and atrophy (Garnier-Crussard et al., 2022). However, the cohort study by Wang et al. (2024) is contrary to this conclusion. Emerging neuroimaging evidence suggests that WMH may serve as a preclinical biomarker. They can predict AD onset at least a decade before clinical symptoms appear (Mortamais et al., 2014).

### Studies on the preclinical phase

An increased volume of greater WMH is associated with a higher number of microhemorrhages in individuals with preclinical AD (Shirzadi et al., 2024). Longitudinal follow-up of cognitively intact individuals over 40 years revealed that those who remained free of AD exhibited distinct neuroimaging profiles characterized by progressive mild cortical atrophy and increasing WMH burden, with more pronounced changes emerging after age 65 (Skampardoni et al., 2024). Amyloid-β (Aβ) deposition significantly accelerates WMH progression, with gender-specific analyses revealing that female participants exhibiting elevated baseline Aβ levels showed significantly greater WMH volume expansion over a 24-months follow-up period (Cha et al., 2024). The author proposes that a rapid WMH increase in the cognitively normal elderly should be treated as a clinical red flag, with gender serving as an integral component of risk assessment models.

### Studies on SCD

Studies have found that subjective cognitive decline promotes the future progression of WMH (Liu et al., 2024). WM volumes of uncinate fasciculus, cingulum, inferior frontooccipital fasciculus, anterior thalamic radiation, and corpus callosum clamp were lower in SCD group than in HC group. However, there were no significant differences in WM lesions number or volume between the SCD and HC groups (Riverol et al., 2024). Differences in the burden of WMH in the brain were observed between patients with positive (SCD+) and negative SCD (SCD−), indicating the possibility of distinct underlying pathologies (Morrison et al., 2023). The bilateral longitudinal superior frontal fasciculus fiber tracts were larger in individuals with SCD compared to those in the HC (Wei et al., 2024). Compared with the HC, patients with SCD had larger temporal, occipital, and frontal WMH, whereas patients with MCI had higher WMH volumes in all regions (Calcetas et al., 2022). The accuracy of diffusion tensor imaging (DTI) in distinguishing SCD from normal controls was 92.68%. Moreover, due to further changes in brain structure and function, the classification accuracy of MCI, AD dementia (d-AD) and normal controls can reach more than 97% (Chen et al., 2023). The author believes that constructing a predictive model integrating WMH distribution patterns and DTI-based fiber tracking metrics could offer critical technical support for early and precise risk stratification of progression from SCD to MCI or d-AD.

### Studies on MCI

Total WMH and regional WMH were increased in MCI and AD patients compared with non-MCI patients. We observed that in all cognitive domains, declines were greater in MCI compared with HC (stronger association between WMH and cognition). However, compared with non-AD patients, the overall cognitive function of AD patients decreased more significantly only in the temporal region. In HC and MCI, we observed strong associations between all cognitive domains of interest and WMH burden, whereas AD patients had only a small number of associations between WMH and overall cognition (Kamal et al., 2023). In HC, higher cognitive reserve (CR) was associated with macromolecular tissue volume (MTV) in the right para-hippocampal cingulate (PHC) and the left superior longitudinal fasciculus (SLF) (Fingerhut et al., 2022). WMHs are associated with cognitive impairment in both patients with MCI and those with AD (van den Berg et al., 2018). Additionally, the MCI group with the presence of vessel amyloidosis had a significant increase in WMH after 5 years of follow-up (Shirzadi et al., 2024). Research utilizing diffusion kurtosis imaging and free water imaging, which effectively differentiated between the MCI, SCD, and HC groups, has identified changes in WM microstructure in individuals with MCI and SCD (Bergamino et al., 2024).

The neurite density index (NDI) of specific WM structures in the bilateral cerebral hemispheres of patients with MCI and AD was significantly decreased, particularly in the bilateral SLF, uncinate fasciculus (UF), left posterior thalamic radiation (PTR), and left cingulate. Conversely, there was a significant increase in the orientation dispersion index (ODI) in WM regions, including the left cingulate, right UF, bilateral PHC, and PTR. Notably, ODI was significantly reduced in the GM of the bilateral hippocampus. Cognitive performance in MCI/AD patients showed a significant correlation with NDI. Microstructural alterations in MCI/AD included decreased fiber directional dispersion in the hippocampus, along with reduced neurite density and increased fiber directional dispersion in specific WM tracts, such as the cingulate, UF, and PTR (Zhong et al., 2023). In MCI, a higher CR was associated with lower MTVs in WM tracts, specifically in the left and right dorsal cingulate gyrus, corpus callosum forceps, right inferior frontooccipital fasciculus, and right SLF (Fingerhut et al., 2022). Patients undergoing cognitive training demonstrated a slower rate of fractional anisotropy decline in multiple WM tracts, particularly in the cingulum-hippocampal pathway, which correlated with improved working memory performance (Gozdas et al., 2024). A critical direction for future research is to further elucidate the correlations between WMH distribution patterns–such as periventricular, deep, and infratentorial–and specific etiologies, including vascular, Aβ-related, and inflammatory pathologies. This can be accomplished through the integration of multimodal neuroimaging–such as amyloid-PET, tau-PET, and high-resolution perfusion imaging–with fluid biomarkers to establish a clinically meaningful etiological classification system for WMH.

### Studies on the dementia phase

Larger global and regional WMH volumes are strongly associated with cognitive decline (Garnier-Crussard et al., 2022). The WMH volume (WMHV) of increased with progressing amyloid and tau pathology in the AD sample. It was found that samples from individuals with AD and MCI exhibited reduced WM volume, and DTI results indicated diminished WM integrity compared to HC (Radanovic et al., 2013). In the early phases of AD, there has been an increase in WMH load, indicating a change in WMH during this period (Pålhaugen et al., 2021). AD shows significantly higher heterogeneity compared to SCD, MCI, or vascular dementia (Roh et al., 2024). In AD, both restricted isotropic diffusion and crossing fibers were reduced, while free water diffusion was elevated in the mesial temporal GM and WM. Restricted isotropic diffusion in the hippocampus decreased more rapidly in participants with AD. Baseline hippocampal limiting isotropic diffusion can predict cognitive decline, and alterations in hippocampal and entorhinal limiting isotropic diffusion are associated with this decline. Additionally, changes in WM and crossing fibers that restrict directional diffusion are linked to memory decline in HC. Microstructural changes in the medial temporal lobe are associated with cognitive decline in prodromal AD, and these changes differ from those observed in normal cognitive aging (Reas et al., 2018). The volume of WMH in the left occipital lobe may be related to the occurrence of delusional AD (Fan et al., 2023).

WMH volume increased with progressing amyloid and tau pathology in the AD sample. Compared with the aβ-negative HC, the aβ-positive AD patients had larger WMHVs in all brain regions, with the largest volume change in the splenium of the corpus callosum (Garnier-Crussard et al., 2022). In the AD sample, the Aβ+T− group showed consistently lower fiber density in most fiber tracts compared to the Aβ−T− HC. The fiber-bundle cross-section was also reduced in the Aβ+T− group. Similarly, the Aβ+T+ group showed lower fiber density and lower fiber-bundle cross-section compared to the Aβ-T− HC. The Aβ+T+ group did not show any additional WM damage regarding fiber density or fiber-bundle cross-section compared to Aβ+T−. In summary, both fiber density and fiber-bundle cross-section were reduced in the presence of amyloid pathology, but not further altered by additional tau pathology. WMHV showed the highest variable importance for fiber density in most fiber tracts, while brain volume showed the highest variable importance for fiber bundle cross-section in all tracts. In simple linear regression analyses, fiber density in the AD sample was likewise associated with WMHV and to some extent with microbleed count but not with lacune count, which was expected given the low number of lacunes and microbleeds in this sample. Fiber density was not associated with brain volume and with age only in selected fiber tracts. Effect sizes for associations with AD PET markers were substantially smaller than with cerebral small vessel disease (SVD) MRI markers. Compared to fiber density, fiber-bundle cross-section was less associated with SVD imaging markers; no significant associations with lacunes or microbleeds. In contrast, fiber-bundle cross-section of all fiber tracts was strongly associated with brain volume and to some extent with age. Associations with AD PET markers were mostly absent or showed only small effect sizes. WM damage represents a critical nexus in the interplay between AD and cerebrovascular disease. Moving forward, it is essential to transcend traditional diagnostic categories and develop multidimensional disease models that incorporate vascular, Aβ, tau, and neural plasticity components. By integrating multimodal neuroimaging, fluid biomarkers, and artificial intelligence, we can advance from a paradigm of “post-symptomatic diagnosis and treatment” toward one emphasizing “early and precise prediction” and “targeted intervention”–ultimately paving the way for delaying or even preventing cognitive decline.

## Longitudinal studies of GM and WM

Alzheimer’s disease progression follows a distinct spatiotemporal pattern of coordinated structural deterioration in both GM and WM (Reas et al., 2018). During the preclinical phase, GM atrophy initiates in the medial temporal lobe–primarily the entorhinal cortex and hippocampus–preceding clinical symptoms by 5–10 years (Platero, 2022), while concurrent WM microstructural alterations, characterized by reduced fractional anisotropy and increased mean diffusivity, become detectable in limbic tracts such as the parahippocampal cingulum and corpus callosum (Mortamais et al., 2014; Skampardoni et al., 2024). As the disease transitions to mild cognitive impairment (MCI), WM degeneration accelerates, frequently surpassing the rate of GM atrophy (Fotenos et al., 2005; Kamal et al., 2023).

**FIGURE 2:**
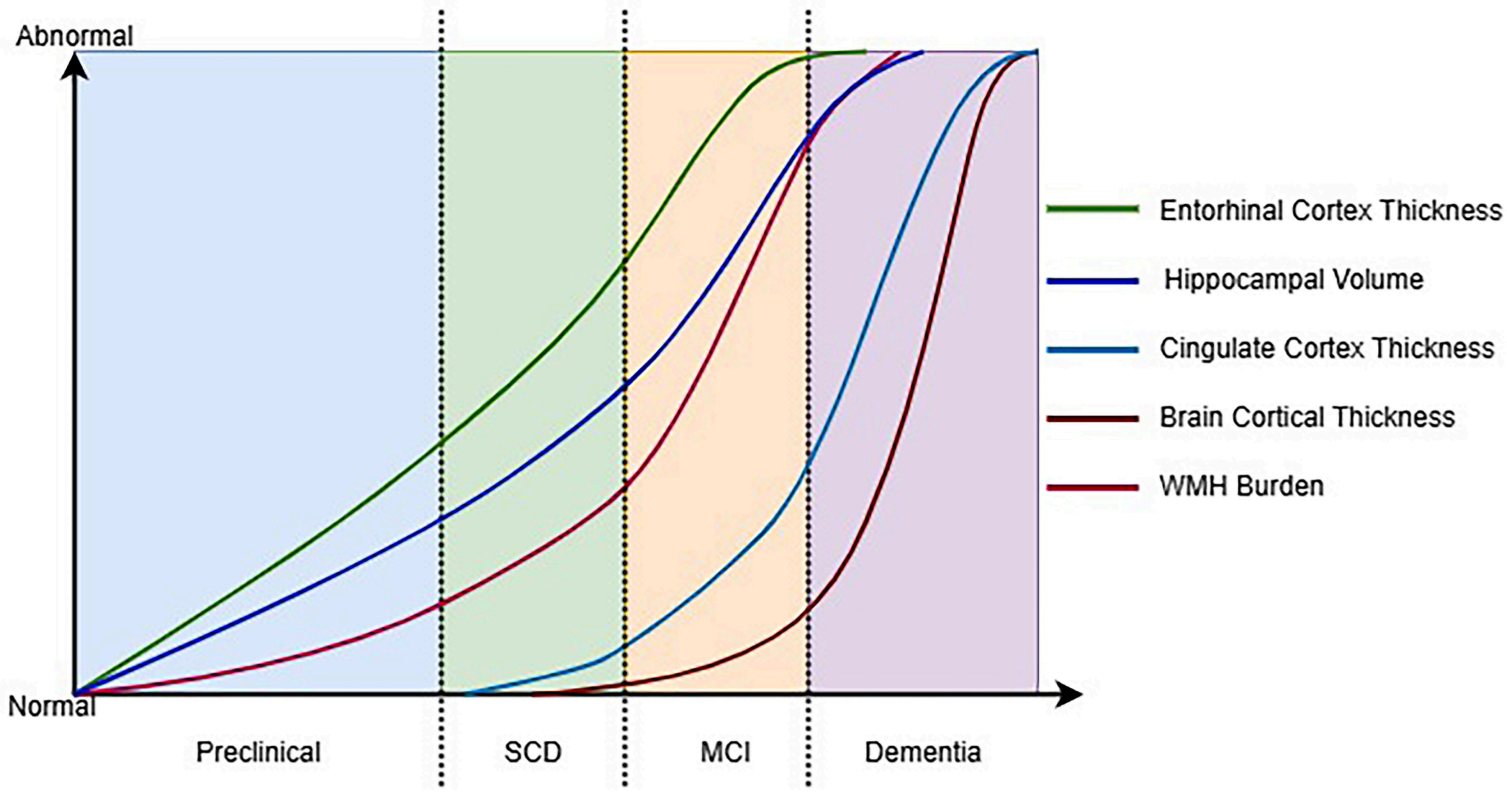
Hypothetical timeline of key structural MRI changes across the AD continuum. SCD, subjective cognitive decline; MCI, mild cognitive impairment; WMH, white matte hyperintensities.

We hypothesize that the progression from intermediate to advanced AD phases demonstrates coupled degeneration between GM and WM. During the intermediate phase, hippocampal atrophy extends to parietal and lateral temporal regions, while corresponding WM damage emerges in association fibers such as the superior longitudinal fasciculus. This coordinated deterioration evolves further in the advanced phase, where widespread cortical thinning develops concurrently with disintegration of major WM tracts, including the cingulum bundle and uncinate fasciculus, suggesting interconnected neurodegenerative mechanisms throughout the disease continuum (Figure 2).

## Discussion

The purpose of this review is to establish a foundation for predicting the risk of progression to AD in patients at various phases, including HC, SCD, and MCI. Additionally, it aims to provide valuable insights to assist clinicians in early diagnosis and the design of effective treatment plans (Lee et al., 2024). Structural MRI data were used to predict the conversion of SCD to MCI or dementia (Lerch et al., 2024). An increasing number of artificial intelligence technologies have been introduced into the diagnosis of degenerative diseases, such as AD (Frizzell et al., 2022; Qiu et al., 2020; Warren and Moustafa, 2023). These advanced technologies not only improve the accuracy of diagnoses but also provide new possibilities for early detection and personalized treatment, driving research and development in this field. Longitudinal studies and large-scale data analysis, such as those from the UK Biobank, are crucial for understanding the genetic and environmental factors influencing AD progression.

### MRI confounding factors

This review synthesizes the rapidly evolving evidence on structural MRI biomarkers in AD. However, a critical challenge is the methodological heterogeneity in the current literature body. Variations in MRI scanner platforms, field strengths, acquisition sequences, and automated segmentation pipelines significantly impact volumetric and microstructural measurements (Song et al., 2024; Tai et al., 2020; Wu et al., 2023). This heterogeneity not only contributes to conflicting results regarding the spatiotemporal sequence of GM and WM changes but also hinders the direct comparison and meta-analysis of findings across cohorts, limiting the generalizability of individual study results. Furthermore, the interpretation of structural changes is complicated by several confounding factors. The frequent co-existence of vascular pathology (e.g., WMH) with AD pathology can obscure the specific signature of AD-related atrophy, particularly in WM metrics (Pålhaugen et al., 2021; Petersen et al., 2024). Factors such as education (Fingerhut et al., 2022; Serra et al., 2022), occupational complexity (Gozdas et al., 2024), and lifestyle (Erickson et al., 2012; Krueger et al., 2025; Pålhaugen et al., 2021) (collectively termed “cognitive reserve”) can modulate the relationship between brain atrophy and clinical symptoms, potentially leading to misclassification of disease phase. Large-scale, multi-center, prospective studies with harmonized MRI protocols and unified analytical methods to ensure cross-cohort reproducibility. Advanced statistical models that can robustly adjust for the confounding factors mentioned above, to isolate the pure AD-related structural trajectory.

### Structural biomarkers in AD frameworks

Against the evolving backdrop of Alzheimer’s disease diagnostic criteria, structural changes in GM and WM have emerged as objective imaging biomarkers of neurodegeneration, progressively weaving their way into the fabric of mainstream diagnostic frameworks (Reas et al., 2018). Within the ATN (Aβ/Tau/Neurodegeneration) classification system, GM atrophy and WM microstructural injury are principally categorized under the “N” domain, supplying critical evidence of neuronal demise to substantiate clinical diagnosis (Jack et al., 2018). Specifically, GM atrophy within the medial temporal lobe–including the entorhinal cortex and hippocampus–has been formally incorporated into the NIA-AA diagnostic criteria as a characteristic neuroimaging hallmark of AD (Heinzinger et al., 2023). Although WM alterations–such as reduced fractional anisotropy in the cingulum bundle and fornix–have not yet been fully enshrined in formal guidelines, they are increasingly recognized as vital elements for disease subtyping and prognostic evaluation, illuminating the earliest disruptions in large-scale neural networks (Chen et al., 2023). Yet, the integration of these structural biomarkers is not without its challenges. First, as representatives of the “N” category, GM and WM alterations exhibit limited specificity; analogous patterns of structural decline may also manifest in vascular cognitive impairment or primary tauopathies, necessitating their interpretation within a multimodal context that incorporates Aβ and tau biomarkers. Second, structural biomarkers and core AD pathologies engage in a complex spatiotemporal dialogue: GM atrophy patterns frequently mirror the topographical spread of tau pathology as captured by Braak staging (Heinzinger et al., 2023), whereas WM injury appears to arise from a confluence of Aβ deposition, vascular compromise, and tau-driven axonal degeneration (Morrison et al., 2023). Moreover, translational efforts are hampered by persistent methodological variability–divergent MRI acquisition protocols, analytical pipelines, and diagnostic thresholds across institutions continue to undermine the reproducibility and broad clinical applicability of GM/WM biomarkers.

### Multimodal AI stratification

The integration of neuroimaging and plasma biomarkers significantly enhances the accuracy of disease staging across the Alzheimer continuum (Dark et al., 2024). The distinct structural and functional alterations identified during preclinical and subjective cognitive decline (SCD) phases offer a potential roadmap for stratifying and targeting high-risk individual (Jobin et al., 2023; Leandrou et al., 2018). Specifically, individuals categorized based on our MRI-based criteria may be directed into a tiered management pathway: those exhibiting isolated default mode network (DMN) functional alterations (preclinical phase) could be enrolled in more frequent cognitive surveillance (Cui et al., 2025), whereas those showing additional WMH may be prioritized for intensive management of vascular risk factors (e.g., hypertension, diabetes) and enrolled in structured lifestyle interventions (Skampardoni et al., 2024).

Looking forward, an AI-augmented clinical workflow cholds significant potential to enhance the early diagnosis and stratification of patients along the Alzheimer’s disease continuum while offering data-driven clinical decision support (Frizzell et al., 2022; Yu et al., 2024). In such a setting, clinicians would upload patient MRI data including T1-weighted, diffusion tensor imaging (DTI), and resting-state functional MRI (rs-fMRI) to an AI platform integrated with the hospital information system. This system would automatically generate a comprehensive report within minutes, quantifying AD risk probability, suggesting a disease phase, and highlighting key abnormal regions–such as entorhinal cortical thinning, hippocampal volume loss, and fornix integrity decline–along with actionable clinical next steps, thereby establishing AI as a powerful tool for auxiliary screening and triage.

### Limitations and future directions

Several limitations in the current literature warrant careful consideration. First, significant methodological heterogeneity exists across studies, including variations in MRI scanner platforms, field strengths, acquisition parameters, and processing pipelines, which directly impact the comparability and reproducibility of structural measurements. Second, the substantial clinical and biological heterogeneity within AD spectrum populations–including differences in age at onset, genetic background, comorbidities (particularly cerebrovascular disease), and cognitive reserve–creates considerable noise that may obscure distinct spatiotemporal patterns of GM/WM alterations. Third, many studies, particularly those focusing on rare subtypes or deep phenotyping, are constrained by limited sample sizes, reducing statistical power for robust subgroup analyses. Fourth, the potential for publication bias toward positive findings may skew our understanding of the true effect sizes and spatial distribution of structural changes. Finally, while our review highlights the promise of integrated biomarkers, the practical implementation of multimodal data fusion presents substantial challenges. These include technical issues in data harmonization, the need for advanced statistical methods capable of handling high-dimensional, non-linear relationships, and the current lack of standardized frameworks for validating and interpreting such integrated models.

## Conclusion

In conclusion, this review synthesizes compelling evidence that the integration of multimodal neuroimaging with clinical assessments and artificial intelligence holds significant potential for transforming Alzheimer’s disease diagnosis and management. The characteristic spatiotemporal patterns of gray and WM alterations across the disease continuum offer valuable biomarkers for early detection, stratification, and progression monitoring. However, the translation of these advanced methodologies into routine clinical practice faces substantial practical challenges that must be acknowledged. These include the significant costs associated with advanced MRI protocols, the pressing need for standardization across imaging platforms and sites, the considerable heterogeneity in patient populations that complicates generalizability, and the limited accessibility of advanced analytical tools in resource-constrained settings. Future efforts must therefore focus not only on technological refinement but also on developing cost-effective, standardized, and accessible implementation frameworks. Only through addressing these translational barriers can the full potential of integrated neuroimaging and AI approaches be realized in diverse clinical contexts, ultimately improving patient care across the Alzheimer’s disease spectrum.
